# Characterization of MicroRNAs and Gene Expression in ACC Oxidase RNA Interference-Based Transgenic Bananas

**DOI:** 10.3390/plants12193414

**Published:** 2023-09-28

**Authors:** Yan Xia, Zhongxiong Lai, Yi-Yin Do, Pung-Ling Huang

**Affiliations:** 1 Institute of Horticultural Biotechnology, Fujian Agriculture and Forestry University, Fuzhou 350002, China; xyfafu@163.com; 2 Department of Horticulture and Landscape Architecture, National Taiwan University, Taipei 10617, Taiwan

**Keywords:** fruit ripening, gene silencing, transcriptome, sRNA-seq, gene regulation

## Abstract

Banana (*Musa acuminata*, AAA group) is a typically respiratory climacteric fruit. Previously, genes encoding ACC oxidase, one of the key enzymes in ethylene biosynthesis, *Mh-ACO1* and *Mh-ACO2* in bananas were silenced individually using RNAi interference technology, and fruit ripening of transgenic bananas was postponed. Here, the differential expression of miRNAs and their targeted mRNAs were analyzed in the transcriptomes of fruits at the third ripening stage, peel color more green than yellow, from the untransformed and RNAi transgenic bananas. Five significantly differentially expressed miRNAs (mac-miR169a, mac-miR319c-3p, mac-miR171a, mac-miR156e-5p, and mac-miR164a-5p) were identified. The predicted miRNA target genes were mainly enriched in six KEGG pathways, including ‘sulfur relay system’, ‘protein digestion and absorption’, ‘histidine metabolism’, ‘pathogenic *E. coli* infection’, ‘sulfur metabolism’, and ‘starch and sucrose metabolism’. After ethylene treatment, the expression of ACC oxidase silencing-associated miRNAs was down-regulated, and that of their target genes was up-regulated along with fruit ripening. The evolutionary clustering relationships of miRNA precursors among 12 gene families related to fruit ripening were analyzed. The corresponding expression patterns of mature bodies were mainly concentrated in flowers, fruits, and leaves. Our results indicated that ethylene biosynthesis is associated with miRNAs regulating the expression of sulfur metabolism-related genes in bananas.

## 1. Introduction

MicroRNAs (miRNAs) are a class of endogenous non-coding RNA with regulatory functions found in eukaryotes, with a size of about 20–24 nucleotides [1]. Mediation of gene expression at the post-transcriptional and translational levels is executed by down-targeting mRNAs or inhibiting gene translation. Significant progress has been made in the study of miRNA function in many species, and previous studies have shown that miRNAs are involved in various metabolic pathways and biological processes in plants, including development, signaling, abiotic stress, and the regulation of symbiotic relationships [2]. miRNAs act as a bridge between transcriptional regulation and fruit maturation hormone responses. In general, the regulatory effects of miRNAs on fruit ripening and aging are either positive or negative. Overexpression of miR172 specifically inhibited *SIAP2a* to enhance ethylene biosynthesis and accelerate fruit ripening [3]. The tomato *rin* mutant fruit does not exhibit ethylene-induced respiratory climacteric phenomenon because no functional RIN binds to the miR172a promoter accumulate miR172 [4]. MiR1971 targets *SlCTR4* splice variants (*SlCTR4sv*), and overexpression of miR1917 enhanced ethylene response and accelerated tomato fruit ripening [5]. For negative regulatory effects, such as overexpression of miR157 delayed tomato fruit ripening by targeting *CNR* (key *SPL* gene for fruit ripening) [6], strawberry fan-miR73 targets abscisic acid insensitive 5 (*ABI5*), which is critical for ABA signaling, to inhibit fruit ripening [7]. The miR164/*SINAC4* component also regulates fruit ripening and carotenoid accumulation [8]. In kiwifruit, miR164 can negatively regulate the target *NAC6*/*7* to participate in fruit ripening [9]. miR319 in tomato negatively regulates tomato fruit flavor by targeting the *TCP* family transcription factor *LANCEOLATE*(*LA*), and overexpression of miR319 in tomato leads to a decrease in soluble sugar content while overexpression of *LA* leads to the opposite result and change in fruit flavor [10]. miR828/858 regulates most R2R3-type MYBs to affect anthocyanin biosynthesis. For example, the accumulation of miR858, a negative regulator of tomato anthocyanin biosynthesis, was negatively correlated with the expression of its target gene *SIMYB7-like*. *SIMYB7-like* transcripts were significantly increased in miR858-silent tomato transgenic plants, leading to a significant accumulation of anthocyanins [11]. Anthocyanin accumulation was increased in overexpressed-miR156 transgenic *Arabidopsis* because one of the miR156 targets, *SPL9*, was down-regulated, the *MYB-Bhlh-WD40* transcriptional activation complex remained stable, and anthocyanin biosynthetic genes were not suppressed; while reducing miR156 activity lead to high levels of flavonols [12].

Several transcription factors involved in fruit ripening have been identified for gene regulation. The wheat TuMYB46L binds to the *TuACO3* promoter through yeast monohybrid and electrophoretic mobility shift assay (EMSA) [13]. In cucumbers, CsWIP1 bound to the promoter of *CsACO2,* one of the five sex-determining genes, and inhibited the expression of *CsACO2*, resulting in suppression of carpel development and enhancement of stamen development [14]. Fone of the five sexes determined with ACC oxidase. In bananas, MaBZR1/2 acts as a transcriptional inhibitor to regulate banana fruit ripening by binding to CGTGT/CG sequences of promoters of ethylene biosynthetic pathway genes *MaACS1*, *MaACO13*, and *MaACO14* [15]. In addition, the functional interaction between chromatin remodeling-related proteins, such as histone H1 (HIS1) and WRKY, was related to banana fruit ripening and stress response [16]. An AGAMOUS-like MADS-box protein *MA-MADS5*, which is mainly bound to the promoter CArG-box, was related to fruit ripening, and its mRNA accumulates abundantly in the pulp of banana fruit [17]. Banana MaMADS36 was directly bound to the *MaBAM9b* promoter *cis*-acting component CA/T(r)G box to increase the transcription level of beta-amylase 9b, thereby accelerating the activity of enzymes and the hydrolysis of starch during fruit ripening [18]. As a suppressor related to the ethylene signaling pathway in banana fruit ripening, MaNAC67-like protein is physically bound to starch degradation-related *MaBAM6*, *MaSEX4*, and *MaMEX1* promoters. It was associated with MaEBF1 to enhance the activities of *MaBAM6* and *MaSEX4* promoters [19].

As a respiratory climacteric fruit, banana fruits produce ethylene massively at the onset of the respiratory climacteric period. An increase in the accumulation of *MACS1* mRNA was concomitant with fruit ripening until Stage 5 [20]. Furthermore, it was found that two ACC oxidase genes, *Mh-ACO1* (GenBank serial number AF030411) and *Mh-ACO2* (GenBank serial number U86045), were involved in banana fruit ripening. At the beginning of fruit ripening, the *Mh-ACO1* gene was highly expressed in the flesh and reached its maximum expression level at the ripening stage 6 [21]. *Mh-ACO2* accumulated from stage 2 of fruit ripening until stage 7 [22]. The expression levels of *Mh-ACO1* and *Mh-ACO2* vary in different tissues and organs of banana fruit [21,22]. To clarify how *Mh-ACO1* and *Mh-ACO2* affect the involvement of miRNAs and their targeted modules in the ripening mechanism of banana fruits, small RNAs of the *Mh-ACO1* and *Mh-ACO2* RNA interference (RNAi) transgenic banana plants (As1 and As2, respectively) [23] were analyzed in this study. We also investigated the expression patterns of selected banana fruit ripening-related miRNAs and target genes at different stages of fruit ripening under different hormone treatments. Moreover, as a key influencing factor for respiratory climacteric changes, it remains to be explored whether any regulatory protein has a synergistic effect on the transcriptional level of *Mh-ACO2* to promote ethylene biosynthesis. Our findings will provide valuable data for in-depth research on the ripening mechanism of banana fruits and lay the foundation for subsequent analysis and research on banana functional genes.

## 2. Results

### 2.1. Acquisition of Total RNA and Distribution of Small RNA Fragment Length in Banana Fruit

**Figure 1:**
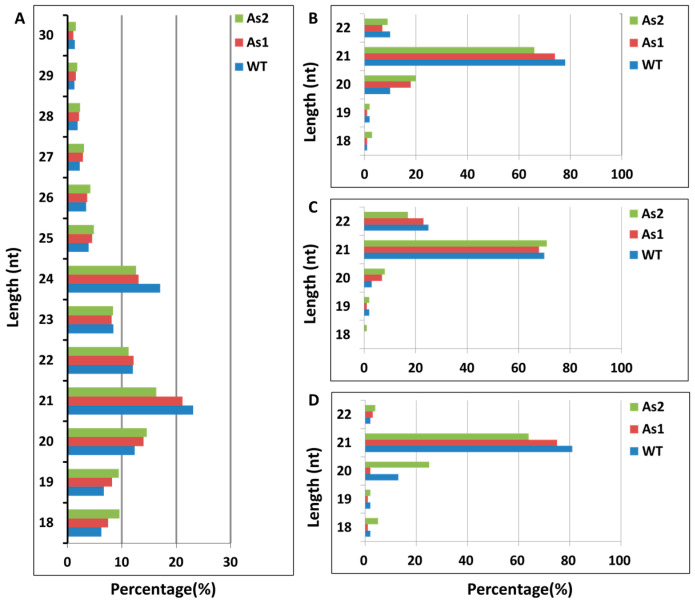
Length distribution of sRNAs in banana ripening fruits. (**A**) Total sRNAs; (**B**) unique sRNAs; (**C**) known miRNAs; (**D**) novel miRNAs. The x-axis is the length of small RNAs, and the y-axis is the percentage of small RNAs with each length. As1, the *Mh-ACO1* RNAi transgenic banana; As2, the *Mh-ACO2* RNAi transgenic banana; WT, untransformed banana.

Quality analysis was conducted on the obtained raw data, and the average GC content in this sequencing was 51.75% under the uniform precursor of the database, which meets the requirements. Statistical analysis of the types and quantities of small RNA obtained by removing connectors, low quality, N bases, and contamination was conducted on the clean sequences. The types, quantities, and percentages of three sample fragments of small RNA from bananas were analyzed (Table 1). The total reads obtained from sequencing in WT, As1, and As2 were 7,663,656, 8,864,286 and 8,402,017, respectively. The clean reads obtained for subsequent analysis after quality control screening were 6,912,621 (90.20%), 8,337,765 (94.06%), and 7,457,309 (88.76%) in WT, As1, and As2, respectively. The length distribution induction analysis found that the general miRNA length concentrated in 21 or 22 nt, most siRNAs were 24 nt long, and piRNAs were more in 28 to 30 nt length. Length distribution (as shown in Table S2) of known and novel miRNAs was 18–22 nt, wherein 21 nt small RNAs were most abundant in three banana samples (Figure 1).

### 2.2. Classification and Annotation of Small RNAs in Wild-Type and RNAi Transgenic Bananas

The total amounts of sRNAs obtained in WT, As1, and As2 were 4,088,721, 4,301,614, and 3,366,260 reads, respectively. The numbers of unique sRNAs obtained from WT, As1, and As2 were 1,356,305, 1,152,324, and 1,160,214, respectively. The unique sRNAs specifically expressed in both As1 and As2 are 160,394 (Table S3). The total small RNA reads of three samples were mapped to the banana genome using Bowtie [24], compared with various RNAs, and summarized the annotation. The successful annotation of WT, As1, and As2 in total sRNA was 2,235,441 (66.41%), 2,755,447 (67.39%), and 3,160,467 (73.47%), respectively. The order of priority in known miRNA, rRNA, tRNA, snRNA, snoRNA, repeat, novel miRNA, and ta-siRNA was adopted to ensure that various small RNAs are mapped to only one annotation. Among them, 121463, 227552, and 41844 sRNAs were identified in WT, As1, and As2 to match known miRNAs, and the types of sRNAs matched to miRNAs in the three samples were 126, 159, and 121, respectively (Table S4). After aligning these sequences with mature miRNA sequences, 25, 26, and 21 miRNAs were identified in the WT, As1, and As2 samples, respectively, resulting in a total of 31 mature miRNAs.

### 2.3. Classification and Annotation of Small RNAs in Wild-Type and RNAi Transgenic Bananas

The total amounts of sRNAs obtained in WT, As1, and As2 were 4,088,721, 4,301,614, and 3,366,260 reads, respectively. The numbers of unique sRNAs obtained from WT, As1, and As2 were 1,356,305, 1,152,324, and 1,160,214, respectively. The unique sRNAs specifically expressed in both As1 and As2 are 160,394 (Table S3). The total small RNA reads of three samples were mapped to the banana genome using Bowtie [24], compared with various RNAs, and summarized the annotation. The successful annotation of WT, As1, and As2 in total sRNA was 2,235,441 (66.41%), 2,755,447 (67.39%), and 3,160,467 (73.47%), respectively. To ensure that various small RNAs are mapped to only one annotation, the order of priority in known miRNA, rRNA, tRNA, snRNA, snoRNA, repeat, novel miRNA, and ta-siRNA was adopted. Among them, 121,463, 227,552, and 41844 sRNAs were identified in WT, As1, and As2 to match known miRNAs, and the types of sRNAs matched to miRNAs in the three samples were 126, 159, and 121, respectively (Table S4). After aligning these sequences with mature miRNA sequences, 25, 26, and 21 miRNAs were identified in the WT, As1, and As2 samples, respectively, resulting in a total of 31 mature miRNAs.

### 2.4. Differential Expression of MicroRNAs among Wild-Type and Two Transgenic Bananas

**Figure 2:**
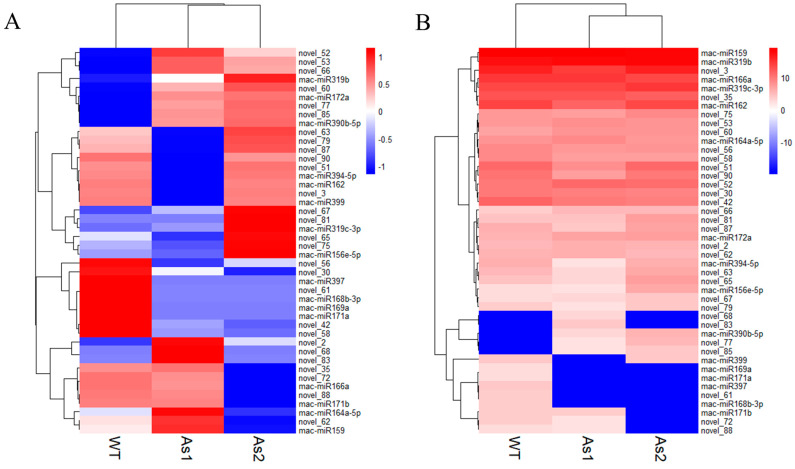
Cluster analysis of differentially expressed miRNAs in small RNA sequencing of RNAi transgenic banana. WT, untransformed banana; As1, the *Mh-ACO1* RNAi transgenic banana; As2, the *Mh-ACO2* RNAi transgenic banana. (**A**): Horizontal axis clustering under processing of Z-score; (**B**): Overall clustering under processing of Z-score.

**Figure 3:**
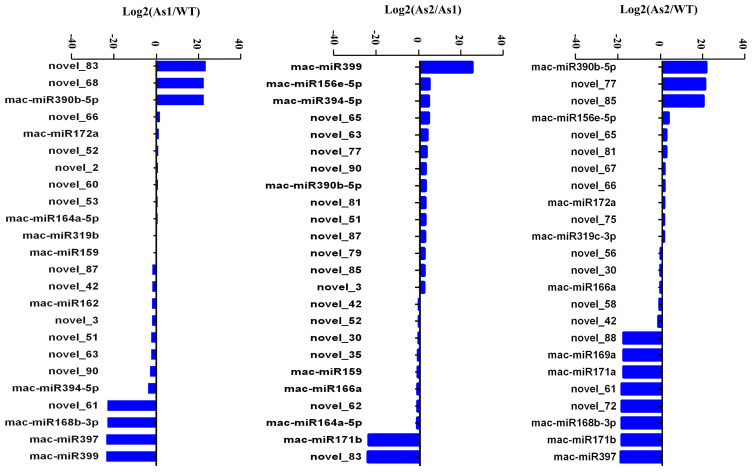
Differential expression of miRNAs in pairwise samples. As1, the *Mh-ACO1* RNAi transgenic banana; As2, the *Mh-ACO2* RNAi transgenic banana; WT, untransformed banana.

A total of 44 miRNAs with significant differences (Table S5) were classified into six groups based on their expression patterns (Figure 2). The results showed that silencing of *Mh-ACO1* and *Mh-ACO2* genes resulted in up-regulation of 7 miRNAs in both transgenic plants, including two known miRNAs (mac-miR172a and mac-miR390b-5p) and five novel miRNAs (novel_52, novel_53, novel_60, novel_66, and novel_85). In addition, silencing of *Mh-ACO1* and *Mh-ACO2* resulted in down-regulation of four miRNAs (mac-miR168b-3p, mac-miR169a, mac-miR171a, and mac-miR397) and four newly discovered miRNAs (novel_42, novel_56, novel_58, and novel_61) expression (Figure 3, Table S5).

Three new miRNAs, novel_2, novel_68, and novel_83, were only up-regulated in *Mh-ACO1* RNAi transgenic banana. Only 9 miRNAs were down-regulated after *Mh-ACO1* gene was silenced, including 3 known miRNAs (mac-miR162, mac-miR394-5p, and miR399) and 6 novel miRNAs (novel_3, novel_51, novel_63, novel_79, novel_87, and novel_90). Only 9 miRNAs were down-regulated after *Mh-ACO2* gene was silenced, including 4 known miRNAs (mac-miR159, mac-miR164a-5p, mac-miR166a, and mac-miR171b) and 5 novel miRNAs (novel_30, novel_35, novel_62, novel_72, and novel_88). Eight miRNAs were only up-regulated in *Mh-ACO2* RNAi transgenic banana, including three known miRNAs (mac-miR156e-5p, mac-miR319c-3p, and mac-miR319b) and five novel miRNAs (novel_65, novel_67, novel_75, novel_77, and novel_81) (Figure 3, Table S5).

### 2.5. Expression Profiles of MicroRNA-Targeted Transcription Factor Genes in Mh-ACO1 and Mh-ACO2 RNAi Transgenic Bananas

**Figure 4:**
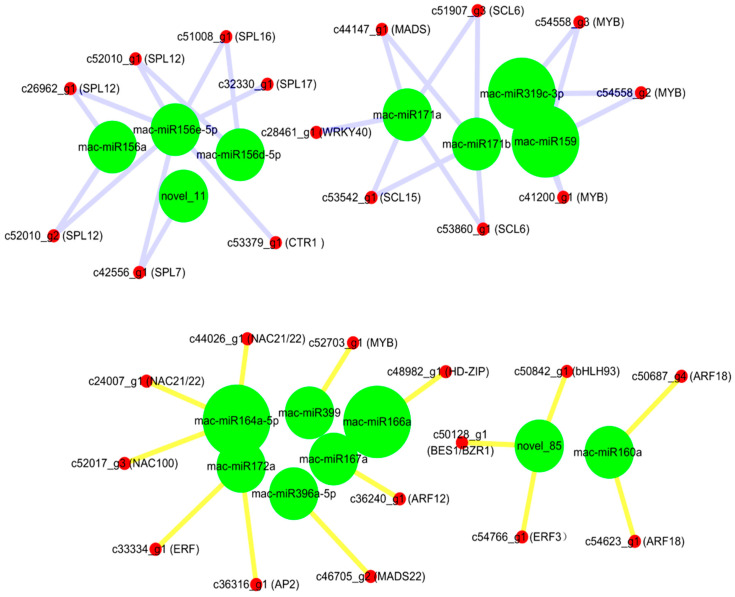
Relationships between miRNAs and their targeted transcription factors.

In this study, transcription factors targeted by all differentially expressed miRNAs were statistically analyzed. Novel_85 targeted the BES1/EZR1 homologous protein 4 (*BEH4*), ethylene-responsive transcription factor 3 (*ERF3*), and *bHLH93*. Auxin response factor 12 (*ARF12*) and auxin response factor 18 (*ARF18*) were targeted by miR167a and miR160a, respectively. Mac-miR172a targeted the floral homologous protein APETALA2 gene (*AP2*) and *ERF*. Mac-miR156a targeted the dual component response regulator *ARR11*, and the miR156 family also targeted the *Squamosa* promoter binding protein-like gene (*SPL*). Mac-miR396a-5p targeted *MADS 22*, while miR171 family members (miR171b, miR171d, miR171e, and miR171a) targeted MADS box protein *AGL62*. Both mac-miR171a and mac-miR171b targeted *scarecrow-like protein 6* (*SCL6*), while the mac-miR171a also targeted *WRKY 40*. MiR164a-5p targeted NAC domain protein 21/22 (*NAC021*) and *NAC100*; mac-miR319-5p targets *WRKY 19*, and mac-miR319c-3p, mac-miR319a, and mac-miR159 all targeted on transcription factor *GAMYB1* (Figure 4).

### 2.6. Feedback Regulation of Sulfur Metabolism Pathway, Ethylene Biosynthesis, and Signaling Pathway in Mh-ACO1 and Mh-ACO2 RNAi Transgenic Bananas

The biological pathways involved with the predicted target genes of the differential miRNAs were analyzed through KEGG (Kyoto Encyclopedia of Genes and Genomes) [25], and KOBAS software version 3.0 [26] was used to identify candidate target genes enriched in the KEGG pathway for statistical analysis. It was found that target genes were mainly enriched in six metabolic pathways: sulfur relay system, protein digestion and absorption, histidine metabolism, pathogenic *Escherichia coli* infection, sulfur metabolism, and starch and sucrose metabolism. The proportion of background reference genes is 7.1%, 6.7%, 3%, 2%, and 2%, respectively.

**Figure 5:**
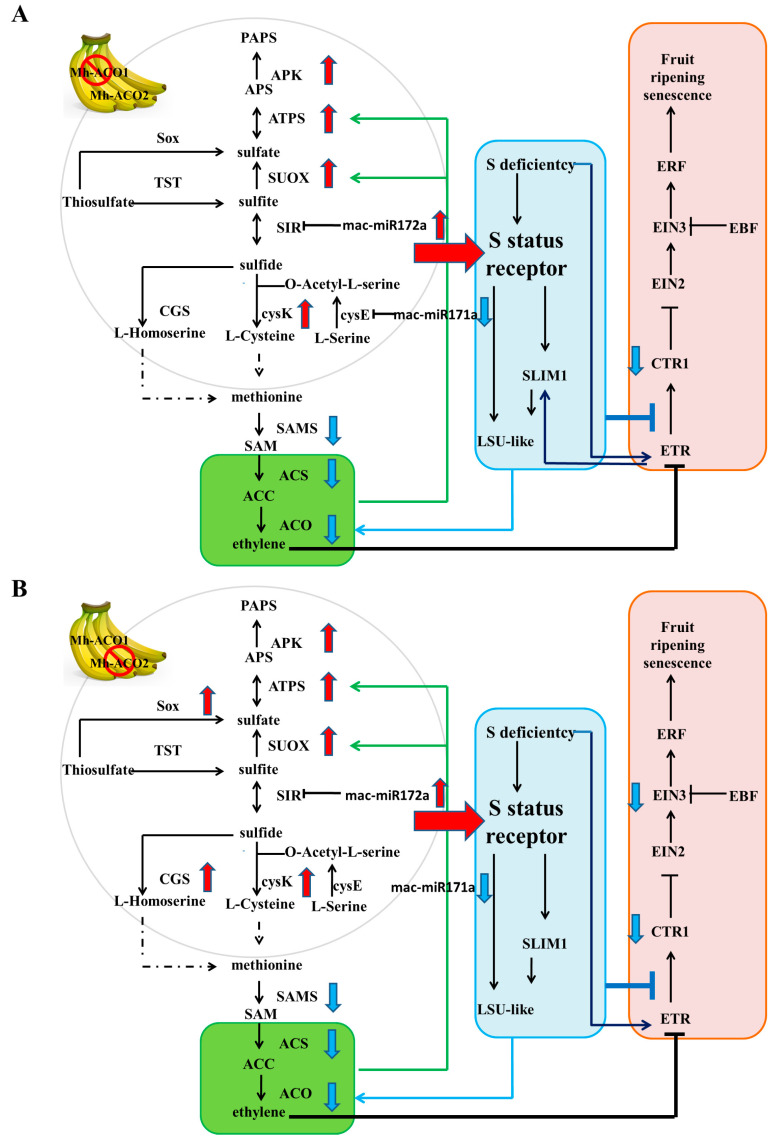
Feedback regulation and expression of miRNAs and their target genes in sulfur metabolism, ethylene biosynthesis, and ethylene signaling pathways in *Mh-ACO1* (**A**) and *Mh-ACO2* (**B**) RNAi transgenic bananas. SAMS, S-adenosylmethionine (SAM) synthetase; ACS, 1-aminocyclopropane-1-carboxylic acid (ACC) synthase; ACO, ACC oxidase; CTR1, serine/threonine-protein kinase Constitutive CTR1; EIN2, ethylene-insensitive protein 2; EIN3, ethylene-insensitive protein 3; ETR, ethylene receptor; ERF, ethylene-responsive transcription factor; EBF, EIN3-binding F-box protein; cysE, serine O-acetyltransferase; cysK, cysteine synthase; SIR, sulfite reductase (ferredoxin); APS, adenosine 5′-phosphosulfate; ATPS, ATP sulfurylase; APK, APS kinase; PAPS, 3′-phosphoadenosine 5′-phosphosulfate; APR, APS reductase; TST, thiosulfate/3-mercaptopyruvate sulfurtransferase; SUOX, sulfite oxidase; CGS, cystathionine gamma-synthase; SOX, L-cysteine S-thiosulfotransferase; SLIM1, sulfur limitation 1; LSU-like, low sulfur up-regulated like protein. Arrowheads with red or blue color indicate differential up- or down-regulation compared with the control WT, respectively.

In the sulfur metabolism pathway, mac-miR172a and mac-miR171a target c29474_g1 and c52353_g1, which correspond to the *SIR* [EC: 1.8.7.1] and *cycE* [EC: 2.3.1.30] genes in sulfur metabolism, respectively. Mac-miR172a was up-regulated in As1-vs-WT and As2-vs-WT with similar folds (Figure 3); the expression of target gene c29474_g1 in As1-vs-WT and As2-vs-WT was down-regulated, and the down-regulation amplitude in As2 was greater than that of As1 (Table S6). These results were consistent with the principle of miRNA inhibition on target gene expression. The expression of mac-miR171a in As1 and As2 was the same as WT and was down-regulated compared to WT; however, the expression of c52353_g1 in As1 and As2 is significantly down-regulated, which is inconsistent with the inhibitory effect of miRNA. The lack of sulfur significantly affects ethylene signaling rather than ethylene yield, and more importantly, recent reports suggest a possible interaction between the Sulfur LIMitation 1 transcription factor (*SLIM1*) and ethylene receptors. In this study, ethylene biosynthesis and ethylene signaling pathways were significantly inhibited due to the silencing of *Mh-ACO1* and *Mh-ACO2* by RNAi technology; however, no miRNA and its target gene enrichment were found in this pathway. It is speculated that the sulfur metabolism pathway may lead to feedback regulation of the maintenance of ethylene levels in an organism under conditions of inhibition of ethylene biosynthesis and signaling after *ACO* gene silencing (Figure 5).

### 2.7. Differential Expression of MicroRNAs and Their Target Genes during Banana Fruit Ripening after Ethylene Treatments

**Figure 6:**
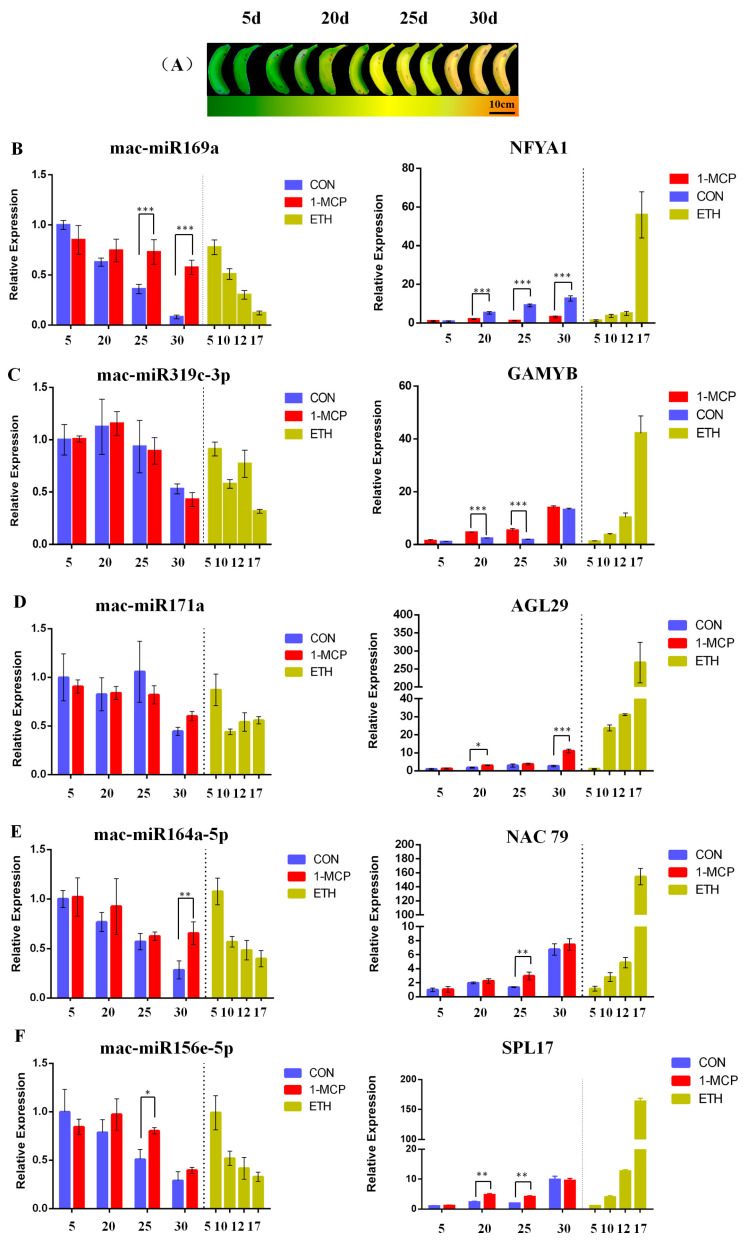
Differential expression analysis of miRNAs and their target genes using qRT-PCR during banana fruit ripening. (**A**) Color changes of banana peel under natural ripening. (**B**–**F**) Relative expression of miRNAs (left) and their target genes (right) during banana fruit ripening after natural ripening (CON), 1-MCP (1-MCP), and ethylene (ETH) treatments. The concentrations of 1-MCP and ethylene applied were 1 and 100 μL·L^−1^, respectively. Samples were collected at stages 1, 3, 5, and 7. The sampling times for the natural ripening and 1-MCP treatment groups were 5 days (stage 1), 20 days (stage 3), 25 days (stage 5), and 30 days (stage 7) after treatments. The sampling time for the ethylene treatment group was 5 days (stage 1), 10 days (stage 3), 12 days (stage 5), and 17 days (stage 7). *, **, and *** indicate significant differences in levels of 0.05, 0.01, and 0.001, respectively.

Five miRNAs (mac-miR169a, mac-miR319c-3p, mac-miR171a, mac-miR156e-5p, and mac-miR164a-5p) and their fruit ripening-related target genes, *NFYA1* [27], *GAMYB* [28], *AGL29* [29], *NAC79* [30], and *SPL17* [31], were analyzed for gene expression by qRT-PCR during fruit ripening after 1-MCP or ethylene treatment (Table S7). According to the overall results, it was found that as the fruit ripening last, all five miRNAs were down-regulated, and ethylene treatment enhanced the down-regulation trend. Under 1-MCP treatment, the trend of decreasing expression levels of these five miRNAs was significantly slowed down as the fruit ripened. The fold change of expression of mac-miR169a between the first and the last ripening stages was larger than that of the other four miRNAs (Figure 6).

Secondly, based on target gene analysis, expression levels of *NFYA1*, *GAMYB*, *AGL29*, *SPL17*, and *NAC79* were investigated. Under ethylene treatment, the expression levels of these five target genes were significantly increased compared to the naturally ripened banana fruits, and the final expression abundance was higher than that of the control group. Under 1-MCP treatment, the expression profiles of these five target genes were significantly lower than those of the naturally ripened banana group. These data indicated that these miRNAs negatively regulated their target genes during banana fruit ripening because the expression levels of these five miRNAs decreased gradually, and expression of their corresponding target genes enhanced concurrently. Moreover, 1-MCP slowed down the decrease in expression levels of miRNAs, and ethylene treatment induced abundant mRNA accumulation of the target genes (Figure 6).

### 2.8. Cloning and Expression Analysis of Banana Pri-mac-miR169a and Pri-mac-miR319c-3p during Fruit Ripening

**Figure 7:**
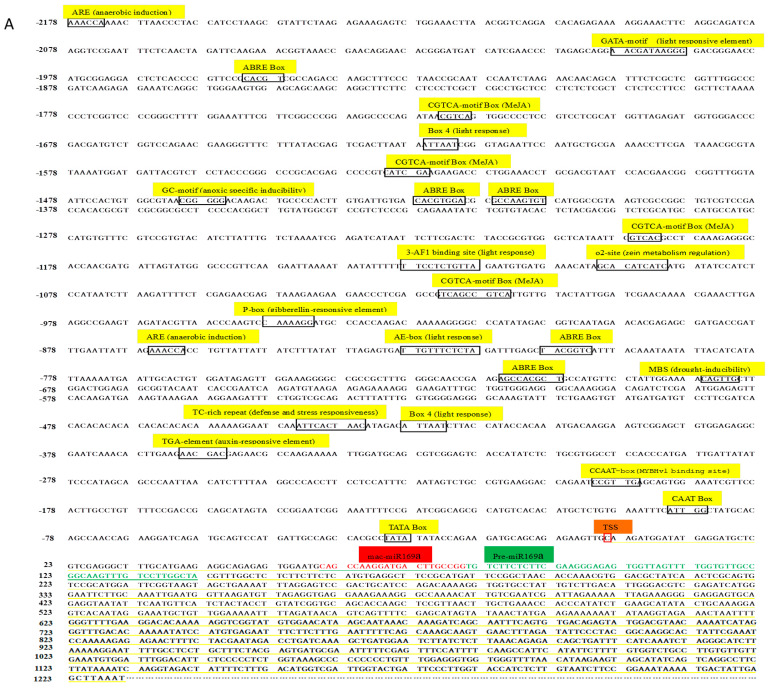

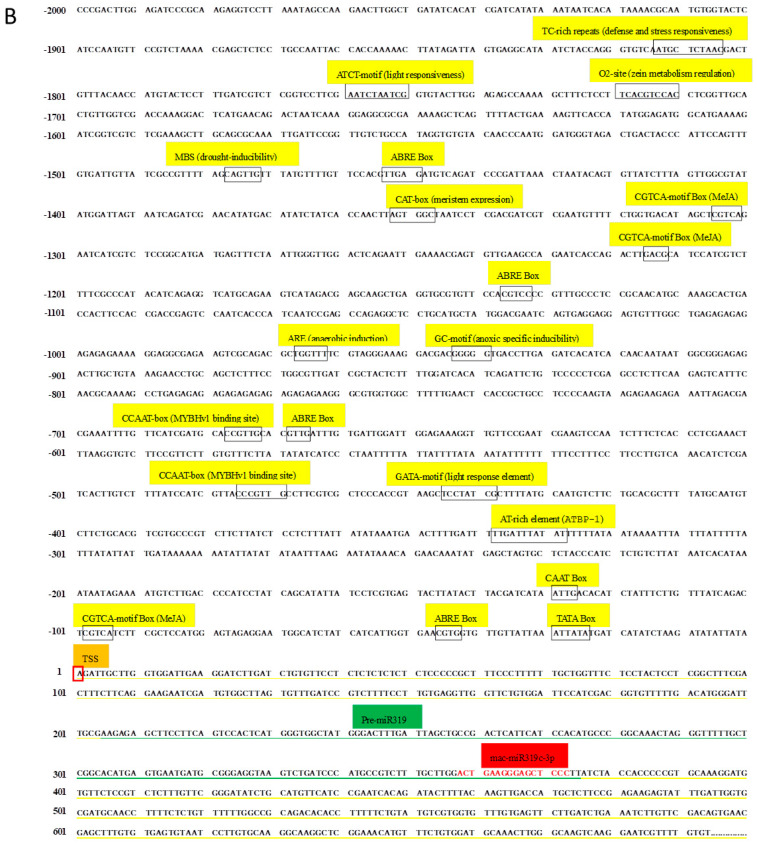
Nucleotide sequences of promoters and genes of *mac-miR169a* (**A**) and *mac-miR319c-3p* (**B**) in banana. *Cis*-acting elements are indicated by black boxes. The red box represents the transcription start site (TSS). Pri-miRNA, pre-miRNA, and mature miRNA are underlined in yellow, green, and red, respectively.

The lengths of mature mac-miR169a and mac-miR319c-3p are both 21 bp, and their predicted precursors are 149 bp and 195 bp, respectively. Based on sequences of miRNA precursors, the cDNAs reverse transcribed from the third stage of banana fruit ripening were used as the templates to perform RACE for cloning 5′- and 3′-end of pri-miRNAs. Finally, the sequence lengths of primary miRNA are 1231 bp and 695 bp, respectively (Figure 7).

After mapping pri-miRNAs to the banana genome, 2 kb sequences upstream to the transcriptional start sites of *pri-mac-miR169a* and *pri-mac-miR319c-3p* were adapted to predict the *cis*-regulatory elements by the Plant CARE website. Abscisic acid response elements (ABRE), MeJA response elements (CGTCA-motif Box), MYB binding elements related to MBS drought regulation, and TGA element auxin response elements were found in both miRNA promoters (Figure 7). However, the *pri-mac-miR169a* promoter has a unique P-box (gibberellin response element), which could not be found in the promoter of *Pri-mac-miR319c-3p* (Figure 7A).

**Figure 8:**
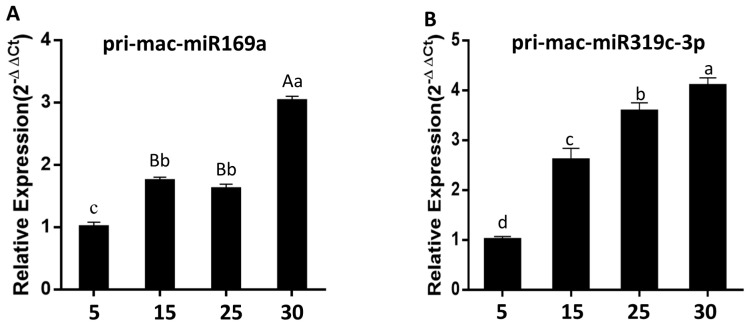
Analysis of the expression of *mac-miR169a* (**A**) and *mac-miR319c-3p* (**B**) in bananas naturally ripen for 5, 15, 25, and 30 d. Different uppercase and lowercase letters indicate significant differences at *p* = 0.01 and *p* = 0.05 levels, respectively.

According to the results of qRT-PCR using pri-miRNA specific primers (Table S8), the expression levels of *Pri-mac-miR169a* and *Pri-mac-miR319c-3p* in bananas showed a significant upward trend with the fruit ripening process. The expression levels of *Pri-mac-miR319c-3p* were significantly higher at each stage of fruit ripening than *Pri-mac-miR169a* under natural conditions. Moreover, *Pri-mac-miR319c-3p* showed a rapid upward trend during fruit ripening stages 1, 3, and 5 and gradually slowed down from stage 5 to stage 7 (Table S9). The expression of *Pri-mac-miR169a* showed a gentle trend during fruit naturally ripening for 15 to 30 d, while it significantly increased at the early stage of fruit ripening (Figure 8).

### 2.9. Intraspecific Cluster Analysis of Mature MicroRNAs Expression in Various Tissues of Bbanana

**Figure 9:**
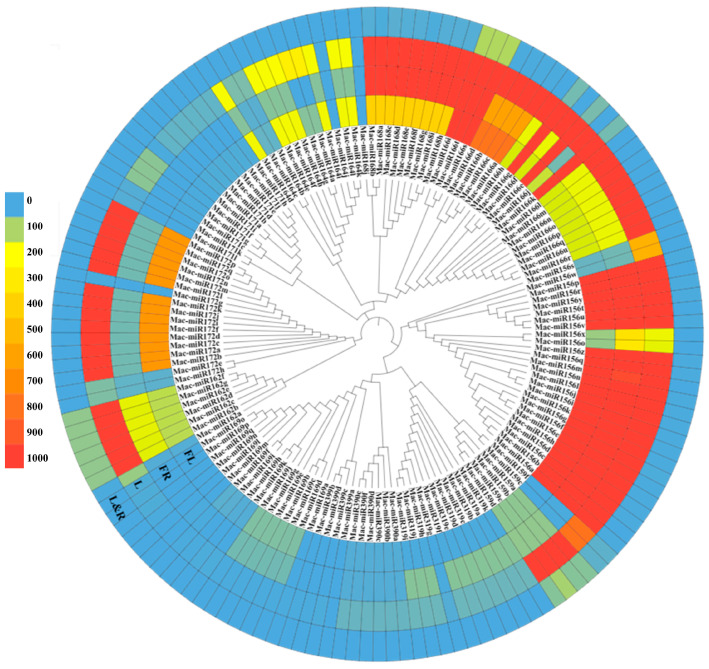
Differential expression and cluster analysis of 12 miRNA gene families related to fruit ripening in various banana tissues. FL, flower; FR, fruit; L, leaf; L&R, leaf and root. Data were adapted from https://www.pmiren.com, accessed on 29 March 2023.

All 12 mature miRNAs related to fruit ripening were identified based on https://www.pmiren.com, accessed on 29 March 2023 [32] (Table S10), and cluster analysis and expression spectrum co-identification analysis were performed. It was found that the gene families with the highest overall expression in fruits were mac-miR168 and mac-miR156, followed by mac-miR162, mac-miR166, mac-miR159 and mac-miR164, and finally mac-miR319, mac-miR169 and mac-miR172. Mac-miR171, mac-miR390, and mac-miR399 had no expression in fruits (Figure 9).

Some specifically expressed miRNAs were found. The expression of these screened fruit-ripening miRNAs is mainly concentrated in flowers, fruits, and leaves, rarely in roots. Mac-miR319 was only highly expressed in fruits and leaves, and its overall expression level was higher than in flowers, leaves, and roots. This indicates that mac-miR319 is mainly involved in banana fruit ripening. Mac-miR171 was only highly expressed in leaves, indicating that mac-miR171 might be mainly involved in the development process of leaves. In the overall analysis, only some members of the mac-miR166 (mac-miR166i, mac-miR166t, and mac-miR166s), mac-miR159 (mac-miR159a, mac-miR159b, mac-miR159c, and mac-miR159d), and mac-miR162 (mac-miR162b-g) gene families were participated in the expression of roots.

Furthermore, six gene families, mac-miR168, mac-miR166, mac-miR156, mac-miR159, mac-miR172, and mac-miR162, were widely expressed in various banana tissues. Normally, members in a general cluster have similar expression spectrums. However, some members in the same gene family were expressed widely differently, such as only 6 out of 17 members of mac-miR169 and only 3 of 12 family members of mac-miR172 were significantly expressed in flowers and leaves, respectively (Figure 9).

## 3. Discussion

Bananas are a typical respiratory climacteric fruit that ripens on its own after picking, making it difficult to control the occurrence of over-ripening and spoilage, reducing the market value of bananas and affecting their economic benefits. Based on the untransformed (WT) and the obtained ACC oxidase RNAi-transgenic banana As1 and As2 [23], this paper carried out miRNA and transcriptome sequencing analysis and identified the regulation mode of related mRNA, miRNA, and target genes at different stages of fruit ripening after the reduction of ethylene biosynthesis in banana through fluorescence quantification, hormone treatments, RACE cloning, and other experimental methods.

The length of the most abundant unique small RNAs, known miRNAs, and novel miRNAs in RNAi transgenic bananas was 21 nt in length (Figure 1), consistent with most plant miRNAs [33]. Among the 44 significantly differentially expressed genes obtained in this miRNA sequencing analysis, the miRNAs mainly expressed at the third stage of banana fruit ripening are mac-miR159, mac-miR319b, mac-miR166a, miR319c-3p, and miR162. Two of these miRNAs (mac-miR319c-3p and mac-miR319b) were significantly up-regulated, and the other two (mac-miR166a and mac-miR159) were significantly down-regulated only in the As2 (Figure 2). Generally, expression of a miRNA is suppressed by ethylene or down-regulated at the late ripening stages if its target gene(s) is an activator for fruit ripening. When *Mh-ACO2* was silenced, less endogenous ethylene was produced to suppress miRNA. So, the product of the target of mac-miR319c-3p, encoding a putative transcription factor GAMYB, might be a positive regulator of ethylene biosynthesis (Table S6). All five miRNAs (mac-miR169a, mac-miR319c-3p, mac-miR171a, mac-miR156e-5p, and mac-miR164a-5p) were down-regulated with a faster trend than that under natural ripening and 1-MCP treatment groups, and their targets were up-regulated (Figure 6). Likewise, expression of *Ade-miR164* in kiwifruit was suppressed by ethylene treatment, and miR164 negatively regulates the targets *NAC6*/*7,* positive regulators of ethylene biosynthesis genes during fruit ripening. The abundance of expressed *Ade-MIR164* showed higher at the early ripening stage and dropped beyond [9]. In tomatoes, there is a similar relationship between miR164 and target *SINAC4* in the regulation of fruit ripening [8]. Oppositely, if the target of a miRNA plays a negative regulator of fruit ripening, the expression of the miRNA is induced by ethylene or consistent with the ripening stages. In *ACO2* RNAi transgenic banana, down-regulation of *mac-miR166a* was suggested that the gene function of the target of mac-miR166a, homeobox-leucine zipper protein HOX9, probably is a suppressor in ethylene biosynthesis (Table S5). In a similar mechanism, overexpressed miR172 enhanced ethylene biosynthesis and accelerated tomato fruit ripening by specifically inhibiting its target *SIAP2a* [3]. Therefore, it is worthy to identify the role of targets of these miRNAs in banana fruit ripening.

The predicted miRNA target genes are mainly enriched in six KEGG pathways, including sulfur transmission system, protein digestion and absorption, histidine metabolism, pathogenic *E. coli* infection, sulfur metabolism, and starch and sucrose metabolism. Interestingly, there was no enrichment of related miRNAs and target genes in the ethylene biosynthesis and signaling pathways. Instead, the expression of upstream sulfur metabolism pathway-related genes in the ethylene synthesis pathway was significantly up-regulated in As1 and As2. In both samples, it was also found that the expression of mac-miR172a was up-regulated, and the expression of *SIR* and *cysE* was suppressed. The other target of mac-miR172a is the ethylene response factor RAP2-7, whose expression was down-regulated only in As1 (Table S5). It is speculated that there is a particular feedback regulation between the sulfur metabolism pathway and ethylene biosynthesis. On the other hand, mac-miR397 was significantly down-regulated in both As1 and As2 banana plants and targets the cytochrome gene P450. At the same time, a newly discovered member of the P450 family, GmCYP82A3, has been proven to participate in jasmonic acid and ethylene signaling pathways [34]. Mac-miR171a targets probable WRKY transcription factor 40 and agamous-like MADS-box protein AGL62. Plant WRKY proteins are mainly accumulated in climacteric pulp and always play an important role in abiotic stress tolerance and regulation of defense response [17]. WRKY also interacts with chromatin remodeling-related proteins, such as connectome protein H1 (MaHIS1), and participates in banana fruit ripening and stress response [16]. The target of mac-miR171a was serine acetyltransferase *SAT5*, which was significantly down-regulated in As1 and As2. The serine acetyltransferase gene family is involved in the committed step of the sulfur assimilation pathway in *Arabidopsis* [35]. As a Cys component required for the synthesis of GSH and SAM, sulfur has impacts on the precursor of ethylene biosynthesis. Cysteine is the final product of S-assimilation and an S compound existing in plants, such as Met, SAM, and S-methylmethionine [Fe/S] clusters. As the main pathway of ethylene biosynthesis, methionine directly controls ethylene metabolism [36].

Mac-miR156e-5p targets Squamosa promoter binding protein-like 7/12/16/17 (SPLs), and SPL7 has been identified as the central regulatory factor for copper homeostasis [37,38]. It has been reported that there is a connection between sucrose signaling and copper accumulation in cells [39]. In addition, protein 1 containing the BSD domain and the homeobox protein BEL1 homolog were also influenced by mac-miR156e-5p. BSD might participate in banana fruit ripening regulation as a transcription activator by directly activating the expression of MaEXP1/2 [40]. Secondly, mac-miR156e-5p also targeted alkaline/acidic invertase, which is involved in starch accumulation in banana fruits. In addition, MaBSD1 participates in banana fruit ripening by directly activating the expression of two ripening-related genes, MaEXP1/2 [40]. Mac-miR164-5p targeted polygalacturonic acid 4-α-galacturotransferase (*Gaut1*) and pectin lyase, a gene involved in the degradation of pectin, leading to disintegration of cell wall structure and fruit softening. Mac-miR169a primarily targets *NFA1*, a gene that may be involved in the appearance of black spots in banana peels. The mac-miR319c-3p target gene transcription factors *GAMYB* were significantly down-regulated in As2. Specific transcription factors such as *GAMYB*, *SPLs*, and *AHBs* are thought to be important regulators involved in fruit development and maturation [41]. These significantly differentially expressed miRNAs and their target genes play a crucial role in the ethylene synthesis and signaling pathways, sugar accumulation, and skin-softening processes of banana fruits.

Mac-miR169a and mac-miR319c-3p, which were significantly down-regulated and up-regulated in As1 and As2, respectively, were chosen for promoter cloning. *Cis*-acting elements responsive to drought and several hormones, such as abscisic acid, MeJA, and auxin, were found in both promoters of miRNA (Figure 7). The promoter of a miRNA is closely related to the tissue, developmental stage, and stress-induced expression specificity of plants, and studying the plant miRNA gene promoter is the molecular basis for revealing miRNA-specific expression [42]. Many promoters of miRNA genes also contain plant hormone response elements and *cis*-elements response to abiotic stresses [43]. In analyzing *Arabidopsis* miRNA promoters, five *cis*-regulatory elements were preferred, including AtMYC2, ARF, SORLREP3, LFY, and TATA box [44]. The excavation of these miRNA promoter elements helps to understand further and explore the differences in miRNA transcriptional expression. It was found that the expression levels of *pri-miR169a* and *pri-miR319c* gradually increased with the fruit ripening process, and their corresponding mature bodies showed opposite expression trends during the fruit ripening. It is speculated that lncRNAs involved in the regulation of fruit ripening upstream of miRNAs act as competitive endogenous RNAs (ceRNAs) to exert the miRNA sponge effect of miRNAs, thereby inhibiting miRNA expression [45]. It is also possible that upstream lncRNAs degrade miRNAs during fruit ripening, resulting in inconsistent expression of pri-miRNAs with miRNAs.

For future work we plan to focus on the construction of overexpression vectors for the selected miRNAs that are crucial for fruit ripening and the use of the optimized banana regeneration system for obtaining a large number of transgenic bananas quickly to deeply explore the regulatory laws of miRNAs and their target genes during fruit ripening.

## 4. Materials and Methods

### 4.1. Plant Materials

*Musa* spp. cv. Pei-Chao (AAA group, Cavendish subgroup) was used as the experimental materials in this research, including untransformed (WT) and *Mh-ACO1* (As1) and *Mh-ACO2* (As2) RNAi transgenic banana plants as previously described [23]. Plants were grown to flower and set fruits under an isolated greenhouse in a planting medium including soil, peat, and sand at a proportion of 1:1:1 by a volume of 300 dm^3^ plastic tank under long-day conditions (16 h light/8 h dark). Fifteen weeks after the first inflorescence emergence, the bananas were harvested when the fruits had developed close to physiological maturity (disappearance of angularity). The wounds of the banana fingers were coated with petroleum grease to prevent contamination and soaked in 400 mg/L of thiamendazim. Each processed fruit was quickly placed in a 4 L bottle and passed through a potassium permanganate solution before introducing fresh air. It was then placed in a distillation flask to remove ethylene and humidity from the air and naturally mature in an environment of 20 °C. During the third stage of fruit maturation, the pulp of each banana finger was frozen in liquid nitrogen and stored at −80 °C for total RNA extraction [46,47]. Banana fruit ripening is classified into stages 1 to 8 by peel color based on the CSIRO Banana Ripening Guide: stage 1, all green; stage 2, green with trace yellow; stage 3, more green than yellow; stage 4, more yellow than green; stage 5, green tip; stage 6, all yellow; stage 7, yellow flecked with brown; and stage 8, yellow with much brown [48]. For miRNA analysis, fruits were harvested from AS1, AS2, and WT at ripening stage 3, the transition stage just before which banana fruits produced large amounts of ethylene.

### 4.2. RNA Isolation and Small RNA Sequencing

The frozen plant tissues were ground to a fine powder with liquid nitrogen, and total RNA from pulp and peel was isolated using the CTAB-based isolation method [49]. The integrity and quality of RNA samples were determined by electrophoresis on a 1.2% agarose gel and OD ratios at A_260_/A_230_ and A_260_/A_280_. The integrated RNA samples comprising equal amounts of RNA from banana fruit peel and pulp were used for small RNA analysis. After ligated with 3′ and 5′ adapters using T4 RNA ligase, the small RNAs were subsequently transcribed to single-stranded cDNA using M-MuLV reverse transcriptase. Next, PCR amplification was performed using ready-to-use NEB’s Longamp Taq 2x Master Mix and primers complementary to adapters. PCR products were purified on an 8% (*w/v*) polyacrylamide gel. After quality was assessed using DNA High Sensitivity Chips, DNA fragments corresponding to 140–160 bp in length, the length of small non-coding RNA plus the 3′ and 5′ adapters, were recovered and dissolved in 8 µL of elution buffer for sequencing. The library was sequenced using Illumina HiSeq^Tm^ 2500 platform, and 50 bp single-end reads were generated. The small RNA tags were mapped to the banana transcriptome by Bowtie [24] with no mismatch for analysis of their expression and distribution on the reference. Mapped small RNA tags were used to look for known miRNA. miRBase 21.0 was used as a reference. According to the characteristics of the hairpin structure of miRNA precursor, novel miRNAs were predicted by mirdeep2 [50] and miREvo [51]. The expression levels of miRNA were estimated by TPM (transcript per million (TPM) normalized in each sample; initial miRNA count × 1,000,000/total count of clean reads) through the following criteria [52]. Differential expression analysis of three samples was performed using the DESeq R package [53]. The corrected *p*-value of 0.05 was set as the threshold for significantly differential expression by default. Expression change was plotted in a heatmap using gplot package (http://cran.r-project.org/web/packages/gplots/index.html, accessed on 28 February 2023) from Bioconductor in R. The target genes for each miRNA were predicted by the criteria of Allen et al. and Schwab et al. for miRNA targets of plants. Identified miRNA targets were aligned against the GO terms in the database (http://www.geneontology.org/, accessed on 28 November 2022), calculating gene numbers for each term, then GO with Corrected *p*-value < 0.05 were significantly enriched in DEGs [54] and KEGG pathway enrichment according to our previously reported approach [25]. Corrected *p*-values smaller than 0.05 were considered as significantly enriched in target gene candidates.

### 4.3. R Language Program for Heat Map Explanation

The graphical visualization of expression profiles of genes/miRNA associated with the expression difference among AS1, AS2, and WT was generated as heat maps with R programming language in the SAS data set of Gplots package (R Development Core Team, 2015).

### 4.4. Chromosome Mapping and Phylogenetic Tree Construction of Banana ACC Oxidase Gene Family

The ACC oxidase gene family chromosome position information and banana chromosome length information were collected and sorted out, and then use MapGene2Chrom [55] (http://mg2c.iask.in/mg2c_v2.0/, accessed on 23 January 2023) to visualize chromosome localization. MEGA7 software [56] was used to elucidate the amino acid sequences of the ACC oxidase gene family and construct the primary evolutionary tree, and then ITOL [57] was used to modify the evolutionary tree.

### 4.5. 1-MCP and Ethylene Treatment of Banana Fruits

Uniform fruits with the highest hardness were transported back to the laboratory on the same day. Single fruit fingers of uniform size and free from pests, diseases, and mechanical damage were selected and surface-disinfected with 0.1% sodium hypochlorite for 10 min. After drying overnight, the fruits were divided into three groups, namely natural ripening (CON), with exogenous ethylene concentration of 99.9% (*w/w*) (ETH), and 0.43% active ingredient 1-MCP (EthylBloc^TM^) (1-MCP). The experimental materials were treated with ten fruit fingers from each group and repeated thrice. The fruits were placed in a polyethylene film bag 0.03 mm thick, and 100 μL·L^−1^ ethylene was injected into the bag as the ETH group. For the 1-MCP treatment group, the powder of 1-MCP was dissolved in distilled water in the amount of 1 μL·L^−1^, sealed in a bottle, and put in a PVC plastic bag tent with banana fruits inside. The reagent bottle caps were immediately opened, and the plastic tents were sealed. Banana fruits ripen naturally and serve as the CON group. Pulps from the middle part of banana fruits at different ripening stages were sampled after treatments for 24 h at 22 °C. Samples were collected at stages 1, 3, 5, and 7. The sampling times for the natural ripening and 1-MCP treatment groups were 5 days (stage 1), 20 days (stage 3), 25 days (stage 5), and 30 days (stage 7) after treatments. The sampling times for the ethylene treatment group were 5 days (stage 1), 10 days (stage 3), 12 days (stage 5), and 17 days (stage 7). Samples were stored at −80 °C after quick freezing by liquid nitrogen.

### 4.6. Analysis of the Expression Levels of MicroRNAs in Various Tissues of Bananas

The expression of miRNA in various tissues of bananas was based on https://www.pmiren.com/, (accessed on 29 March 2023) Collect website login data [32], and MEGA was used to output raw Newick data. The website ITOL was reused to undergo evolutionary tree modification and heat map setting [57].

### 4.7. Real-Time Fluorescence Quantitative PCR

Sample collection and RNA extraction of pulp from stages 1, 3, 5, and 7 of banana fruit were performed using the High Purity Total RNA Extraction Kit (R1002, SinoGene, Beijing, China). Thermo First cDNA Synthesis Kit (Q1010, SinoGene, China) and One-Step miRNA RT Kit (Q1014, SinoGene, Beijing, China) were used to reverse transcribe cDNAs from mRNAs and miRNAs, respectively. Primers used for mRNAs and miRNAs are listed in Tables S10 and S11, respectively. The PCR conditions were: 95 °C initial denaturation step for 10 min; 40 cycles for 95 °C denaturation for 20 s, 60 °C annealing for 30 s, and 95 °C for 15 s; 60 °C for 30 s; and a final 15 s dissociation step of 95 °C. The relative expression levels were calculated by the 2^−ΔΔCT^ method. Firstly, the expression levels in the Ct values of candidate genes were homogenized with reference to the *ACTIN* gene and then normalized with the WT Ct mean to obtain the standardized expression levels of banana fruits. Each experiment has three biological replicates and three technical replicates.

## 5. Conclusions

This paper mainly explores the impact of *Mh-ACO1* and *Mh-ACO2* gene silencing on the regulatory network related to banana fruit ripening. Based on the previously established banana *Mh-ACO1* and *Mh-ACO2* silenced transgenic banana plants (As1 and As2, respectively), miRNA sequencing was performed in the fruits of As1, As2, and WT at the third ripening stage, and five significantly differentially expressed miRNAs were identified. Using ethylene and 1-MCP treatment, as well as RACE cloning technology, the regulatory patterns of its primary miRNAs, mature miRNAs, and their target genes during banana fruit ripening were discovered. According to cluster analysis, the family members in a general cluster have similar functions. Their expression profiles are basically consistent, mainly in flowers, fruits, and leaves. In conclusion, this study deeply excavated the key regulatory network of *ACO* regulating fruit ripening and provides a new idea for the subsequent analysis of banana fruit ripening.

## Figures and Tables

**Table 1:** Summary of data filtering in banana small RNAs.

Sample	WT	As1	As2
total_reads	7,663,656 (100.00%)	8,864,286 (100.00%)	8,402,017 (100.00%)
N% > 10%	899 (0.01%)	296 (0.00%)	1190 (0.01%)
low quality	381 (0.00%)	712 (0.01%)	7365 (0.09%)
5_adapter_contamine	4189 (0.05%)	2226 (0.03%)	2212 (0.03%)
3_adapter_null or insert_null	737,544 (9.62%)	513,274 (5.79%)	924,855 (11.01%)
with ployA/T/G/C	8022 (0.10%)	10,013 (0.11%)	9086 (0.11%)
clean reads	6,912,621 (90.20%)	8,337,765 (94.06%)	7,457,309 (88.76%)

Numbers in parentheses are the percentages in total reads.

## Data Availability

Data sharing is not applicable to this article.

## Supplementary Materials

The following supporting information can be downloaded at: https://www.mdpi.com/article/10.3390/plants12193414/s1, Table S1: Length distribution in WT, *ACO1* and *ACO2* silenced transgenic banana plants; Table S2: Statistics of common and unique sequences in WT, *Mh-ACO1* (As1) and *Mh*-*ACO2* (As2) RNAi transgenic banana plants; Table S3: The statistical of unique small RNA in WT, *Mh-ACO1* (As1) and *Mh*-*ACO2* (As2) RNAi transgenic banana plants matching with various types of small RNAs; Table S4: Differential expression of miRNAs among WT, As1 and As2; Table S5: Expression of miRNA-targeted genes in RNAi transgenic bananas; Table S6: Relative abundance of related miRNAs and their target genes based on qRT-PCR data analysis under hormone treatment; Table S7: Nucleotide sequences of primers used qRT-PCR for detection of pri-miRNAs; Table S8: Relative abundance of pri-miRNAs based on qRT-PCR data analysis during banana fruit ripening; Table S9: Statistics of miRNA expression in different tissues of banana based on https://www.pmiren.com; Table S10: Primer design for detection of miRNA target genes during banana fruit ripening; Table S11: Primer design for detection of miRNAs during banana fruit ripening.
