# Supplementary material for: Characterization of MicroRNAs and Gene Expression in ACC Oxidase RNA Interference-Based Transgenic Bananas
Source: Plants (Basel). 2023 Sep 28;12(19):3414. doi: 10.3390/plants12193414 (PMC10574930; doi:10.3390/plants12193414)
Supplement: Supplementary file 1 [file plants-12-03414-s001.zip › Table_S1.pdf]

Table S1. Length distribution of sRNAs in WT, *ACO1* and *ACO2* silenced transgenic banana plants.

Sample / length (nt)	WT		As1		As2	
	reads	percentage	reads	percentage	reads	percentage
Unique sRNA						
18	2105	0.011594	3356	0.012385	2100	0.033999
19	2928	0.016127	2012	0.007425	1214	0.019655
20	18064	0.099491	47504	0.175303	12297	0.199087
21	140738	0.775143	200216	0.738854	40854	0.661421
22	17729	0.097646	17894	0.066034	5302	0.085839
Known-miRNA						
18	1825	0.015025	3216	0.014133	1894	0.045263
19	2020	0.016631	1430	0.006284	869	0.020768
20	16309	0.134271	44561	0.195828	10607	0.253489
21	98695	0.812552	170604	0.749736	26631	0.636435
22	2614	0.021521	7741	0.034019	1843	0.044045
Novel-miRNA						
18	280	0.004659	140	0.003224	206	0.010340
19	908	0.015108	582	0.013401	345	0.017317
20	1755	0.029201	2943	0.067764	1690	0.084827
21	42043	0.699539	29612	0.681833	14223	0.713899
22	15115	0.251493	10153	0.233778	3459	0.173618
