# Supplementary material for: Characterization of MicroRNAs and Gene Expression in ACC Oxidase RNA Interference-Based Transgenic Bananas
Source: Plants (Basel). 2023 Sep 28;12(19):3414. doi: 10.3390/plants12193414 (PMC10574930; doi:10.3390/plants12193414)
Supplement: Supplementary file 1 [file plants-12-03414-s001.zip › Table_S10.pdf]

Table S10. Primer design for detection of miRNA target genes during banana fruit ripening.

Gene Name	Primer	Primer Sequence(5'-3')	Size of PCR product (bp)
NAC79	Forward	TCGATCGGGTTACTGGAAAG	129
	Reverse	ACCCAATCGGTTTTCCTACC	
AGL29	Forward	CCGACCAGCTCCTACAACAG	100
	Reverse	ATGTCGCCTTCGTTCTTGAC	
GAMYB	Forward	GCCATTCCACACCAACTCTT	145
	Reverse	GAGCCGCTTAGTACCTGTGC	
SPL17	Forward	AGCCAACAACCCATTCAGTC	128
	Reverse	GTTGGTTGTCCCTCCTGCTA	
NYA1	Forward	GAGACGCAGAGTGCAGACAG	116
	Reverse	AGTTCCAGCTGGGTATGTGG	
Actin	Forward	TTAATCCCAAAGCGAACAGG	93
	Reverse	AGAGGACAGCCTGGATAGCA	
