# Supplementary material for: Characterization of MicroRNAs and Gene Expression in ACC Oxidase RNA Interference-Based Transgenic Bananas
Source: Plants (Basel). 2023 Sep 28;12(19):3414. doi: 10.3390/plants12193414 (PMC10574930; doi:10.3390/plants12193414)
Supplement: Supplementary file 1 [file plants-12-03414-s001.zip › Table_S11.pdf]

Table S11. Primer design for detection of miRNAs during banana fruit ripening

miRNA Name	Primer Sequence（5'-3')
miR156e-5p	TGACAGAAGAGAGTGAGCAC
miR164a-5p	TATATGGAGAAGCAGGGCA
miR169a	TATATCAGCCAAGGATGACTTGC
miR171a	TGATTGAGCCGTGCCAATATC
miR319	ACAATTGGACTGAAGGGAGCT
U6 fwd primer	ATTGGAACGATACAGAGAAG
