# Supplementary material for: Characterization of MicroRNAs and Gene Expression in ACC Oxidase RNA Interference-Based Transgenic Bananas
Source: Plants (Basel). 2023 Sep 28;12(19):3414. doi: 10.3390/plants12193414 (PMC10574930; doi:10.3390/plants12193414)
Supplement: Supplementary file 1 [file plants-12-03414-s001.zip › Table_S2.pdf]

Table S2. Statistics of common and unique sequences in WT, *Mh-ACO1* (As1) and *Mh-ACO2* (As2) RNAi transgenic banana plants.

| Sample | Unique sRNAs | Percentage | Total sRNAs | Percentage |
|---|---|---|---|---|
| WT | 1,356,305 | 36.97% | 4,088,721 | 34.78% |
| As1 | 1,152,324 | 31.41% | 4,301,614 | 36.59% |
| As2 | 1,160,214 | 31.62% | 3,366,260 | 28.63% |
| WT&As1 | 172,439 | 7.38% | 5,906,549 | 70.40% |
| As1-specific | 979,885 | 41.94 | 1,130,828 | 13.48% |
| WT-specific | 1,183,866 | 50.68% | 1,352,958 | 16.13% |
| WT&As2 | 152,723 | 6.46% | 4,818,817 | 64.64% |
| As2-specific | 1,007,491 | 42.62% | 1,253,095 | 16.81% |
| WT-specific | 1,203,582 | 50.92% | 1,383,069 | 18.55% |
| As1&As2 | 160,394 | 7.45% | 5,304,154 | 69.17% |
| As1-specific | 991,930 | 46.09% | 1,132,664 | 14.77% |
| As2-specific | 999,820 | 46.46% | 1,231,056 | 16.05% |
| As1 U As2 U WT | 3,323,965 | 100.00% | | |
| As1 &As2 & WT | 140,678 | 4.23% | | |
| As1- specific | 960,169 | 28.89% | | |
| As2- specific | 987,775 | 29.72% | | |
| WT- specific | 1,171,821 | 35.25% | | |
