# Supplementary material for: Characterization of MicroRNAs and Gene Expression in ACC Oxidase RNA Interference-Based Transgenic Bananas
Source: Plants (Basel). 2023 Sep 28;12(19):3414. doi: 10.3390/plants12193414 (PMC10574930; doi:10.3390/plants12193414)
Supplement: Supplementary file 1 [file plants-12-03414-s001.zip › Table_S3.pdf]

Table S3. The statistical of unique small RNA in WT, *Mh-ACO1* (As1) and *Mh-ACO2* (As2) RNAi transgenic banana plants matching with various types of small RNAs.

	WT		As1		As2	
Types	no.	%	no.	%	no.	%
known_miRNA	126	0.03	159	0.04	121	0.03
rRNA	19089	4.89	20744	4.87	21397	5.46
tRNA	1	0.00	0	0.00	1	0.00
snRNA	760	0.19	867	0.20	1011	0.26
snoRNA	460	0.12	521	0.12	524	0.13
repeat	832	0.21	834	0.20	549	0.14
novel_miRNA	505	0.13	475	0.11	334	0.09
ta-siRNA	221	0.06	246	0.06	129	0.03
other	368647	94.37	401702	94.40	368022	93.86
total	390641	100.00	425548	100.00	392088	100.00
