# Supplementary material for: Characterization of MicroRNAs and Gene Expression in ACC Oxidase RNA Interference-Based Transgenic Bananas
Source: Plants (Basel). 2023 Sep 28;12(19):3414. doi: 10.3390/plants12193414 (PMC10574930; doi:10.3390/plants12193414)
Supplement: Supplementary file 1 [file plants-12-03414-s001.zip › Table_S4.pdf]

Table S4. Differential expression of miRNAs among WT, As1 and As2.

miR-name	WT	As1	As2
mac-miR156e-5p	5.646878	3.740079	81.47038
mac-miR159	412916.7	540531.2	284608.6
mac-miR162	12519.13	3149.147	12155.38
mac-miR164a-5p	265.4033	437.5893	195.5289
mac-miR166a	22977.15	21643.84	10591.15
mac-miR168b-3p	11.29376	0.000001	0.000001
mac-miR169a	5.646878	0.000001	0.000001
mac-miR171a	5.646878	0.000001	0.000001
mac-miR171b	11.29376	11.22024	0.000001
mac-miR172a	50.8219	123.4226	130.3526
mac-miR319b	172314.5	228956.4	300218.3
mac-miR319c-3p	11169.52	12170.22	25549.11
mac-miR390b-5p	0.000001	7.480159	48.88223
mac-miR394-5p	56.46878	3.740079	65.1763
mac-miR397	16.94063	0.000001	0.000001
mac-miR399	16.94063	0.000001	16.29408
novel_2	39.52815	71.06151	48.88223
novel_3	70648.09	17529.75	71954.64
novel_30	1072.907	729.3155	521.4104
novel_35	6234.153	6429.197	3584.697
novel_42	3184.839	852.7381	668.0571
novel_51	1886.057	362.7877	2069.348
novel_52	903.5005	1877.52	1466.467
novel_53	175.0532	302.9464	276.9993
novel_56	479.9846	168.3036	244.4111
novel_58	400.9283	145.8631	130.3526
novel_60	124.2313	220.6647	260.7052
novel_61	11.29376	0.000001	0.000001
novel_62	50.8219	67.32143	32.58815
novel_63	39.52815	7.480159	81.47038
novel_65	22.58751	7.480159	130.3526
novel_66	11.29376	41.14087	32.58815
novel_67	5.646878	7.480159	16.29408
novel_68	0.000001	7.480159	0.000001
novel_72	11.29376	3.740079	0.000001
novel_75	169.4063	134.6429	391.0578
novel_77	0.000001	3.740079	32.58815
novel_79	11.29376	3.740079	16.29408
novel_81	22.58751	22.44048	130.3526
novel_83	0.000001	14.96032	0.000001
novel_85	0.000001	3.740079	16.29408
novel_87	67.76254	18.7004	97.76445
novel_88	5.646878	3.740079	0.000001
novel_90	1197.138	157.0833	1026.527
