# Supplementary material for: Characterization of MicroRNAs and Gene Expression in ACC Oxidase RNA Interference-Based Transgenic Bananas
Source: Plants (Basel). 2023 Sep 28;12(19):3414. doi: 10.3390/plants12193414 (PMC10574930; doi:10.3390/plants12193414)
Supplement: Supplementary file 1 [file plants-12-03414-s001.zip › Table_S5.pdf]

Table S5. Expression of miRNA-targeted genes in RNAi transgenic bananas.

miRNA	Target Gene	WT	As1	As2	NR description
mac-miR172a	c29474_g1	22.94	13.00	9.51	SIR
	c33334_g1	3.40	0.00	5.09	floral homeotic protein APETALA 2-like
mac-miR156e-5p	c32330_g1	1.73	1.35	0.20	SPL17
	c71850_g1	0.00	3.31	0.00	alkaline/neutral invertase CINV2-like
mac-miR164a-5p	c44026_g1	0.79	2.90	7.54	NAC domain-containing protein 79
	c24007_g1	0.00	2.35	0.25	NAC domain-containing protein 21/22-like
	c52017_g3	4.9	0.00	0.00	NAC domain-containing protein 100-like
mac-miR169a	c52656_g2	13.76	7.67	8.15	putative Nuclear transcription factor Y subunit A-1
mac-miR168b-3p	c41242_g1	41.21	3.18	15.00	uncharacterized protein LOC103992069
	c42571_g2	2.30	1.96	2.71	cytoplasmic tRNA 2-thiolation protein 2
	c44639_g1	0.07	3.74	3.13	leucine-rich repeat receptor-like protein FASCIATED EAR2
	c46744_g1	13.13	143.05	86.02	uncharacterized protein LOC103984436
mac-miR171a	c44147_g1	2.36	27.38	43.40	putative Agamous-like MADS-box protein AGL29
	c52353_g1	56.92	12.76	18.18	cycE
mac-miR319c-3p	c41200_g1	3.03	1.05	0.79	putative Transcription factor GAMYB
mac-miR162	c21676_g1	2.08	0.14	0.52	uncharacterized protein LOC103976854
mac-miR166a	c48982_g1	13.38	40.20	12.76	homeobox-leucine zipper protein HOX9
mac-miR390b-5p	c46309_g1	11.95	16.84	15.59	sorting nexin 2B-like
mac-miR397	c29475_g1	3.65	0.00	0.00	laccase-22-like
	c39516_g1	0.00	0.09	6.85	laccase-22-like
mac-miR171b	c44147_g1	2.36	27.38	43.40	agamous-like MADS-box protein AGL62
mac-miR394-5p	c30884_g1	78.57	29.47	5.00	tubulin beta-7 chain
	c34590_g1	3.12	7.08	2.73	F-box/kelch-repeat protein At1g23390-like
	c39482_g1	1.16	2.38	1.31	zinc finger protein NUTCRACKER-like
	c48278_g2	80.03	88.08	23.88	BURP domain-containing protein 3-like
	c48800_g1	15.25	9.23	8.41	peptide chain release factor 1, mitochondrial
novel_85	c42169_g2	9.00	12.23	8.88	UDP-glucuronic acid decarboxylase 4
	c39780_g1	7.49	1.28	1.24	Exo-poly-alpha-D-galacturonosidase
	c51322_g1	61.91	220.76	267.68	Beta-amylase 1
	c48267_g2	32.63	41.07	9.38	Endoglucanase 9
