# Supplementary material for: Characterization of MicroRNAs and Gene Expression in ACC Oxidase RNA Interference-Based Transgenic Bananas
Source: Plants (Basel). 2023 Sep 28;12(19):3414. doi: 10.3390/plants12193414 (PMC10574930; doi:10.3390/plants12193414)
Supplement: Supplementary file 1 [file plants-12-03414-s001.zip › Table_S6.pdf]

Table S6. Relative abundance of related miRNAs and their target genes based on qRT-PCR data analysis under hormone treatment.

Gene name	Ripening stage	CON	Ethylene	1-MCP
miR156e-5p	1	0.9999999±0.231184	0.9905604±0.176831	0.8428439±0.07994946
	3	0.7869563±0.1307947	0.5196584±0.07412175	0.9730312±0.1612713
	5	0.509716±0.1023105	0.4163609±0.1125588	0.8035975±0.03187547
	7	0.2888147±0.09189472	0.3293833±0.04726039	0.3952406±0.03137211
miR164a-5p	1	1±0.08739998	1.07627±0.135255	1.020322±0.193167
	3	0.7684343±0.09550255	0.5695545±0.05357159	0.9254171±0.2814901
	5	0.5719554±0.08269076	0.4845915±0.09886383	0.9254171±0.04164499
	7	0.2848911±0.09117351	0.3987537±0.08343399	0.6567959±0.115017
miR169a	1	0.9999999±0.04475325	0.7768515±0.07397803	0.8508651±0.1435644
	3	0.6285105±0.04099707	0.5096691±0.05285799	0.745111±0.1126414
	5	0.3609061±0.04606939	0.3025351±0.04303856	0.729654±0.1230125
	7	0.08166129±0.01888612	0.1214609±0.01919406	0.5758334±0.07168743
miR171a	1	0.9999999±0.2410049	0.871148±0.1620488	0.9058298±0.06689071
	3	0.8254373±0.169655	0.4397493±0.02951873	0.8392494±0.06740341
	5	1.058162±0.3142746	0.5412126±0.09614668	0.8209224±0.09363046
	7	0.4451036±0.0412968	0.5596281±0.03752429	0.6018702±0.04717654
miR319a	1	1±0.146107	0.9123567±0.06621674	1.006989±0.03017835
	3	1.12404±0.2634942	0.5777582±0.04116603	1.155121±0.1138454
	5	0.9351863±0.2503944	0.7703611±0.1296697	0.8937243±0.1268178
	7	0.5306573±0.04720258	0.316552±0.01784366	0.4288501±0.06659437
SPL17	1	1±0.2252764	1.07155±0.2162052	1.134669±0.3142523
	3	2.311682±0.5209848	3.990681±0.7992577	4.754861±0.6763011
	5	1.925321±0.2003287	12.64841±0.8615631	4.095992±0.5560142
	7	9.86064±1.94484	163.501±8.858391	9.496761±1.267264
NAC79	1	1±0.2508764	1.147418±0.3471828	1.051698±0.4207808
	3	1.970744±0.1299981	2.799208±0.6332215	2.253409±0.3147966
	5	1.365222±0.06395751	4.865106±0.7409787	2.955648±0.5653102
	7	6.747022±0.8047503	154.5816±11.66036	7.432178±0.8329742
NYA1	1	1±0.3055323	1.283211±0.3972303	0.8363353±0.303381
	3	2.029987±0.2046173	3.698874±0.813805	5.328921±0.6704891
	5	1.196399±0.1114529	5.016406±1.038418	9.154362±0.7446067
	7	3.169845±0.4944974	55.95711±11.92657	12.72574±1.370186
AGL29	1	1±0.3125947	1.032785±0.176102	1.245582±0.307687
	3	1.768646±0.2106106	23.77768±1.674184	3.014783±0.2206557
	5	2.897509±0.8667189	31.12359±0.4484213	3.741688±0.4914555
	7	2.704027±0.4603983	267.5995±56.11243	11.05473±1.052026
GAMYB	1	0.9999999±0.2193118	1.153303±0.2860011	1.35658±0.5192871
	3	2.311048±0.2284263	3.667512±0.5216252	4.57862±0.177711
	5	1.78818±0.2986197	10.25722±1.720167	5.325398±0.7499726
	7	13.19274±0.5731872	42.17392±6.552601	13.94018±0.7492861
