# Supplementary material for: Characterization of MicroRNAs and Gene Expression in ACC Oxidase RNA Interference-Based Transgenic Bananas
Source: Plants (Basel). 2023 Sep 28;12(19):3414. doi: 10.3390/plants12193414 (PMC10574930; doi:10.3390/plants12193414)
Supplement: Supplementary file 1 [file plants-12-03414-s001.zip › Table_S7.pdf]

Table S7. Nucleotide sequences of primers used qRT-PCR for detection of pri-miRNAs.

Primer name	Primer sequences (5’→3’)
5’adaptor Primer	GTTTTCCCAGTCACGACACGTAACGGCATGACAGTGGGGGGGGGGGG
3RACE adaptor	GTTTTCCCAGTCACGACACGTAACGGCATGACAGTGTTTTTTTTTTTTTTTTTT
RACEOuter	GTTTTCCCAGTCACGACACGTAAC
3RACEInner	GACACGTAACGGCATGACAGTG
169-5race5race invertase	CAGCTACTTACCGAATCCATG
169-5raceR1	AGCCGGAATCATGCGGAAGCCTCACATG
169-5raceR2	AAGACACCGGCAAGTCATCCTTGGCTGC
169-3raceF1	ATGCAGCCAAGGATGACTTGCCGGTGTC
169-3raceF2	ACGCTATCAACTCGCAGTGTCCGCATGG
319-5race invertase	CTCGGAAGAGCATGGTCAAC
319-5raceR1	GGAGAACACATCCTTTGCACGGGGGTGG
319-5raceR2	GCCGGGCATGTGGATGAATGAGTCGGCA
319-3raceF1	GAGCTTCCTTCAGTCCACTCATGGGTGG
319-3raceF2	GCCGACTCATTCATCCACATGCCCGGCA
