# Supplementary material for: Characterization of MicroRNAs and Gene Expression in ACC Oxidase RNA Interference-Based Transgenic Bananas
Source: Plants (Basel). 2023 Sep 28;12(19):3414. doi: 10.3390/plants12193414 (PMC10574930; doi:10.3390/plants12193414)
Supplement: Supplementary file 1 [file plants-12-03414-s001.zip › Table_S8.pdf]

Table S8. Relative abundance of pri-miRNAs based on qRT-PCR data analysis during banana fruit ripening

Pri-miRNA	Ripening stage	Relative expression
Pri-miR169a	1	1.000±0.134228
	3	1.740403±0.1055411
	5	1.61055±0.1336337
	7	3.025719±0.1277512
Pri-miR319c-3p	1	0.9999998±0.117999
	3	2.596222±0.417377
	5	3.576351±0.300469
	7	4.088816±0.273541
