# Supplementary material for: Characterization of MicroRNAs and Gene Expression in ACC Oxidase RNA Interference-Based Transgenic Bananas
Source: Plants (Basel). 2023 Sep 28;12(19):3414. doi: 10.3390/plants12193414 (PMC10574930; doi:10.3390/plants12193414)
Supplement: Supplementary file 1 [file plants-12-03414-s001.zip › Table_S9.pdf]

Table S9. Statistics of miRNA expression in different tissues of banana based on https://www.pmiren.com.

mature_miRNA_ID	Flower	Fruit	Leaf	Leaf and Root
Mac-MIR156a	3068.594	4584.687	12674.15	3.635103
Mac-MIR156b	1147.873	1143.919	4819.938	1.801773
Mac-MIR156c	3068.594	4584.687	12674.15	3.635103
Mac-MIR156d	3068.594	4584.687	12674.15	3.635103
Mac-MIR156e	3036.023	4404.834	12318.11	3.436156
Mac-MIR156f	3036.023	4420.775	12308.92	4.735211
Mac-MIR156g	3036.023	4404.834	12318.11	3.436156
Mac-MIR156h	3068.594	4584.341	12673.94	3.635103
Mac-MIR156i	3031.37	4401.023	12254.71	3.331332
Mac-MIR156j	3031.37	4401.023	12254.71	3.331332
Mac-MIR156k	3068.594	4584.687	12674.15	3.635103
Mac-MIR156l	3068.594	4584.341	12673.94	3.635103
Mac-MIR156m	1112.045	962.6804	4435.61	1.801773
Mac-MIR156n	3036.023	4404.834	12318.11	3.436156
Mac-MIR156o	75.37713	304.6062	170.6103	0.230504
Mac-MIR156p	3068.594	4584.687	12674.15	3.635103
Mac-MIR156q	3036.023	4404.834	12318.11	3.436156
Mac-MIR156r	1147.873	1143.919	4819.938	1.801773
Mac-MIR156s	27.45216	37.77256	543.4625	0.125679
Mac-MIR156t	3068.594	4584.687	12674.15	3.635103
Mac-MIR156u	1147.873	1143.919	4819.938	1.801773
Mac-MIR156v	3068.594	4584.687	12674.15	3.635103
Mac-MIR156w	27.45216	37.77256	543.4625	0.125679
Mac-MIR156x	80.49533	334.4084	178.472	0.052412
Mac-MIR156y	3031.37	4401.023	12254.71	3.331332
Mac-MIR156z	3036.023	4404.834	12318.11	3.436156
Mac-MIR159a	67.93248	71.38667	3977.793	61.67445
Mac-MIR159b	67.93248	71.38667	3977.793	61.67445
Mac-MIR159c	0.930582	2.079223	802.0256	19.48362
Mac-MIR159d	67.93248	71.38667	3977.793	61.67445
Mac-MIR159e	67.46718	71.38667	3945.542	95.17003
Mac-MIR159f	0.930582	2.079223	802.0256	19.48362
Mac-MIR162a	11.63227	9.703042	79.39467	2.367606
Mac-MIR162b	116.3227	162.8725	2427.322	70.31942
Mac-MIR162c	117.2533	163.5656	2427.256	70.31942
Mac-MIR162d	120.9756	168.7636	2442.248	70.62319

Table S9 (cont.)

mature_miRNA_ID	Flower	Fruit	Leaf	Leaf and Root
Mac-MIR162e	120.9756	168.7636	2442.248	70.62319
Mac-MIR162f	120.9756	168.7636	2442.248	70.62319
Mac-MIR162g	116.3227	163.219	2425.894	70.26701
Mac-MIR164c	24.19513	14.20803	46.69707	0.052412
Mac-MIR164d	182.394	59.6044	191.9006	0.670658
Mac-MIR164e	187.047	61.33709	192.5232	0.314474
Mac-MIR164f	187.047	61.33709	192.5232	0.314474
Mac-MIR164g	59.09195	0.693074	272.0048	0.314474
Mac-MIR164h	182.394	59.95094	194.1666	0.314474
Mac-MIR164i	182.394	59.6044	191.9006	0.670658
Mac-MIR164j	0.465291	0.346537	2.905605	0
Mac-MIR164k	187.047	61.33709	192.5232	0.314474
Mac-MIR164l	182.394	59.6044	191.9006	0.670658
Mac-MIR166a	797.0434	602.9747	6568.748	2.870874
Mac-MIR166b	796.5781	602.6282	6563.411	3.876861
Mac-MIR166c	797.0434	602.6282	6568.583	2.870874
Mac-MIR166d	797.0434	602.9747	6568.748	2.870874
Mac-MIR166e	6049.247	7922.187	51008.96	44.95547
Mac-MIR166f	150.289	177.4271	4401.395	1.655238
Mac-MIR166g	5918.501	7882.335	47238.82	40.03036
Mac-MIR166h	151.2196	177.4271	4411.014	1.602826
Mac-MIR166i	5914.313	7762.78	46638.26	107.9641
Mac-MIR166j	133.5385	41.58447	3799.402	1.141818
Mac-MIR166k	5918.501	7882.335	47238.82	40.03036
Mac-MIR166l	151.2196	177.4271	4411.014	1.602826
Mac-MIR166m	151.2196	177.4271	4411.014	1.602826
Mac-MIR166n	151.2196	177.4271	4411.014	1.602826
Mac-MIR166o	151.2196	177.4271	4411.014	1.602826
Mac-MIR166p	151.2196	177.4271	4411.014	1.602826
Mac-MIR166q	151.2196	177.4271	4411.014	1.602826
Mac-MIR166r	151.2196	177.4271	4411.014	1.602826
Mac-MIR166s	5914.313	7762.78	46638.26	107.9641
Mac-MIR166t	5912.917	7761.047	46634.15	107.0003
Mac-MIR166u	151.2196	177.4271	4411.014	1.602826
Mac-MIR168a	402.9419	1735.805	1923.396	4.034649
Mac-MIR168b	403.8725	1756.251	1961.345	11.90445
Mac-MIR168c	407.5949	1762.488	1990.579	12.31304
Mac-MIR168d	403.8725	1756.251	1961.345	11.90445
Mac-MIR168e	403.8725	1756.251	1961.345	11.90445

Table S9 (cont.)

mature_miRNA_ID	Flower	Fruit	Leaf	Leaf and Root
Mac-MIR168h	403.8725	1756.251	1961.345	11.90445
Mac-MIR168f	402.9419	1735.805	1923.396	4.034649
Mac-MIR168g	403.8725	1756.251	1961.345	11.90445
Mac-MIR168i	403.8725	1756.251	1961.345	11.90445
Mac-MIR168j	0	1.039612	0.830091	0
Mac-MIR169a	0.930582	0.693074	2.050213	0
Mac-MIR169b	52.11258	38.46563	3.94324	0
Mac-MIR169c	51.182	37.77256	3.94324	0
Mac-MIR169d	51.64729	38.11909	3.94324	0
Mac-MIR169e	52.11258	38.46563	3.94324	0
Mac-MIR169f	14.42402	2.079223	2.166717	0.125679
Mac-MIR169g	52.11258	38.46563	3.94324	0
Mac-MIR169h	9.305819	1.386149	4.00955	0
Mac-MIR169i	13.95873	1.732686	2.166717	0.125679
Mac-MIR169j	52.11258	38.46563	3.94324	0
Mac-MIR169k	9.305819	1.732686	4.00955	0
Mac-MIR169l	0.930582	0.693074	1.817859	0
Mac-MIR169m	1.861164	2.079223	0.415086	0
Mac-MIR169n	1.395873	1.386149	2.921885	0
Mac-MIR169o	1.395873	0.693074	7.029831	0
Mac-MIR169p	1.395873	0.693074	7.029831	0
Mac-MIR169q	1.861164	1.039612	7.926314	0.125679
Mac-MIR172a	652.8032	39.50524	4063.14	1.393728
Mac-MIR172b	652.8032	39.50524	4063.14	1.393728
Mac-MIR172c	652.8032	39.50524	4063.14	1.393728
Mac-MIR172d	652.8032	39.50524	4063.14	1.393728
Mac-MIR172e	16.75047	23.56453	51.67827	0
Mac-MIR172f	652.8032	39.50524	4063.14	1.393728
Mac-MIR172g	1.395873	1.039612	3.735778	0.314474
Mac-MIR172h	16.75047	23.56453	51.67827	0
Mac-MIR172i	652.8032	39.50524	4063.14	1.393728
Mac-MIR172j	652.8032	39.50524	4063.14	1.393728
Mac-MIR172k	652.8032	39.50524	4063.14	1.393728
Mac-MIR172l	652.8032	39.50524	4063.14	1.393728
Mac-MIR172m	652.8032	39.50524	4063.14	1.393728
Mac-MIR172n	654.6643	39.85178	4079.329	1.498552
Mac-MIR172o	652.8032	39.50524	4063.14	1.393728
Mac-MIR172p	652.8032	39.50524	4063.14	1.393728
Mac-MIR172q	652.8032	39.50524	4063.14	1.393728

Table S9 (cont.)

mature_miRNA_ID	Flower	Fruit	Leaf	Leaf and Root
Mac-MIR319a	3.257036	60.64401	30.89128	0.461008
Mac-MIR319b	3.257036	60.64401	30.89128	0.461008
Mac-MIR319c	3.257036	60.64401	30.89128	0.461008
Mac-MIR319d	3.257036	60.64401	30.89128	0.461008
Mac-MIR319e	3.257036	60.64401	30.89128	0.461008
Mac-MIR319f	3.722327	60.29747	33.13219	0.461008
Mac-MIR319g	0.930582	2.772298	9.461952	0.230504
Mac-MIR319h	3.722327	60.29747	33.13219	0.461008
Mac-MIR319i	3.722327	60.29747	33.13219	0.461008
Mac-MIR319j	3.722327	60.29747	33.13219	0.461008
Mac-MIR319k	3.722327	60.29747	33.13219	0.461008
Mac-MIR390a	8.375237	8.316893	26.6638	0.251359
Mac-MIR390b	8.375237	8.316893	26.6638	0.251359
Mac-MIR390c	8.375237	8.316893	26.6638	0.251359
Mac-MIR390d	8.375237	8.316893	26.6638	0.251359
Mac-MIR390e	8.375237	8.316893	26.6638	0.251359
Mac-MIR390f	8.375237	8.316893	26.6638	0.251359
Mac-MIR399a	0.465291	0.346537	1.112068	0
Mac-MIR399b	0	0.346537	2.09119	0
Mac-MIR399c	0.930582	0.693074	4.35707	0.377038
Mac-MIR399d	0	0.346537	2.09119	0
Mac-miR171a	6.514072957	0.693074419	22.9536494	0.335328818
Mac-miR171b	6.514072957	0.693074419	22.95365	0.335328818
Mac-miR171c	3.722327404	0.34653721	65.43455	0.681360431
Mac-miR171d	6.514072957	0.693074419	22.95365	0.335328818
Mac-miR171e	6.514072957	0.693074419	22.95365	0.335328818
Mac-miR171f	3.722327404	0.34653721	65.43455	0.681360431
Mac-miR171g	3.722327404	0.34653721	65.43455	0.681360431
Mac-miR171h	6.514072957	0.693074419	22.95365	0.335328818
Mac-miR171i	2.326454627	1.386148838	2.124973	0.052412341
Mac-miR171j	6.514072957	0.693074419	22.95365	0.335328818
Mac-miR171k	0	0	9.311632	0
Mac-miR171l	0	0	9.311632	0
